# The past, present, and future of peaking thermal power plants in the United States

**DOI:** 10.1016/j.isci.2026.114886

**Published:** 2026-02-02

**Authors:** Max Vanatta, Brian Sergi, Wesley Cole, Paul Denholm, Trieu Mai

**Affiliations:** 1 National Laboratory of the Rockies, Golden, CO, USA

**Keywords:** energy management, energy Resources, energy systems

## Abstract

Today’s power systems rely on “peaker plants” to reliably serve load during peak demand periods. In this study we consider the present competitiveness of different peaking options, how much and what kinds of plants have provided U.S. peaking capacity, and potential future peaking fleet compositions. We explore how capital intensity impacts the breakeven capacity factor between two potential resources: combustion turbines (CTs) and combined cycle plants (CCs). CTs outcompete CCs below 12%–17% annual capacity factor at today’s prices, but can shift with changes to fuel or start costs. Historically, gas CTs and petroleum steam plants most closely fit the role of peakers. Peaking capacity could grow from approximately 280 GW today to 460–770 GW in 2050 composed of a wider range of resources. We conclude by discussing implications of this shift, with a focus on the potential planning considerations for shifting to a greater utilization of CCs for peaking needs.

## Graphical abstract

**Figure:**
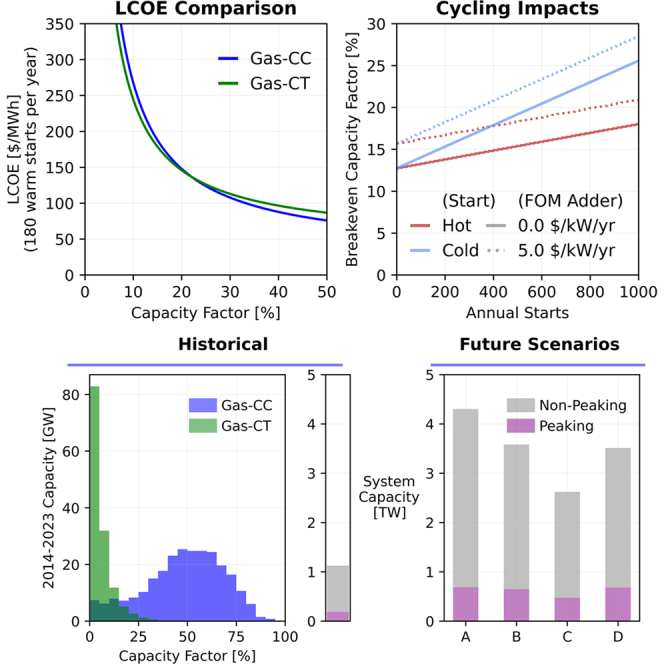

## How do peaking power plants fit within the power system?

Peaker plants have been—and likely will be—needed to meet high demand hours and provide the “backup” capacity of the grid.

Power system planners and operators are charged with maintaining their systems with a high level of reliability. One aspect of that challenge includes ensuring that there is sufficient generating capacity to meet demand at all times (referred to as resource adequacy^1^). Historically, planners have addressed this need by planning to meet their maximum, or peak, demand plus a reserve margin. Because peak demand (or near-peak demand) levels occur relatively infrequently, meeting demand during these time periods requires generation capacity that is only needed infrequently. The general result is that a large portion of the generation fleet has a relatively low utilization rate, or capacity factor. For example, using data from the seven U.S. independent system operators, Mai et al.^2^ showed that 35%–50% of the total capacity is only needed to meet the highest 10% of electricity demand.

With increasing deployment of wind and solar, meeting peak demand has extended to that of meeting “net peak,” or the maximum demand periods after generation from variable renewable energy is accounted for. Given the spatial and temporal patterns of wind and solar, which span sub-hourly to interannual timescales in their variability, this transition is likely to increase the need for peaking resources.^2^^,^^3^ In a study of options for decarbonizing the U.S. by 2035, Denholm et al.^4^ found that, in an “All Options” scenario 21% of total capacity, primarily fossil generators, was used to supply only 4% of the annual energy. These generators must offset their emissions using bioenergy carbon capture and sequestration or direct air capture, increasing their effective costs. The existence and use of these generators in the least-cost system—even with those additional costs for offsetting their emissions—indicates they still provided substantial benefits to the system. Previous work has similarly shown that having generation technologies that can address net peaking needs can greatly reduce system costs.^5^^,^^6^

The definition and characteristics of a peaking power plant are not clearly delineated. Colloquially, plants were typically considered as belonging to one of three categories: baseload, intermediate (load-following), and peaking. Baseload plants were plants that had the least flexibility—either due to technical constraints or economics—and were run continuously, whereas intermediate plants would ramp up and down regularly to manage load fluctuations and serve intra-day demand shifts while remaining online during that period. Peakers were those plants used the least, turning on only to meet the highest net demand unable to be met with cheaper sources, such as diurnally or for only a few hours a year. Although these generation archetypes are generally referenced in industry, research, and government alike, the distinctions between the roles are not formally defined. Recent deployment of storage and generation resources such as wind and solar have challenged these traditional categorizations as these technologies do not easily fit into the standard definitions. Furthermore, even though these categories are often associated with specific plant types, they represent functional roles, and individual power plants in a system can sometimes operate according to multiple roles.

With uncertain load growth and diverse cost, technological readiness, and feasibility of peaker plant options, the optimal composition of the future peaking plant fleet is an open question. Further adding to the planning challenge is the viability of currently installed power plants that have not historically been used as peakers shifting their operations to fill this role. While models can dictate that these operational changes to thermal power plant yield the lowest cost system, it is less known if these models rely on the most accurate assumptions for these assets with non-conventional behavior.

In this work, we consider the present, past, and future of thermal peaking power plants in the U.S. with the intention of highlighting the discrepancy between past operation and the projections in long term planning models. We first examine the question of what makes a cost-effective peaker by highlighting how capital and variable costs influence the capacity factor at which natural gas combustion turbines and combined cycle units are competitive with each other. Using historical data, we next examine which power plants have traditionally operated as peakers. We then draw from previous modeling work to estimate future peaking capacity and the types of resources that tend to be favored for meeting those needs based on long-term modeling. Finally, we qualitatively discuss how the discrepancies between past and future power system compositions may highlight potential areas where peaking plant representation needs to be improved.

## What makes a peaker plant cost-effective?

The low utilization characteristic of peaker plants lends the role toward low capital cost technologies, but changing factors such as increasing variable generation, evolving load profiles, and policies could increase the impact of plant efficiency, fuel costs, and flexibility for choosing the right peaker technology.

In the U.S., peakers have typically been needed to meet demand during hot summer afternoons and evenings, or during periods of extreme cold in the winter. Increasingly, peakers might be needed seasonally to operate continuously for multiple days during times of low wind and solar output or periods of sustained high load.

To be economical at relatively low utilization, the most competitive peaking resources are typically ones with low capital costs.^2^ To illustrate this, we compare the economics of two potential peaking options, namely combustion turbine (CT) and combined-cycle (CC) plants, at different levels of utilization. Although combined cycle plants are more efficient than combustion turbines, they are more capital intensive, making them less competitive as capacity factors decrease. For each plant type we calculate the levelized cost of electricity (LCOE) using cost assumptions from the 2024 annual technology baseline^7^ (method described in SI and parameters in Table S1). Although LCOE has limitations for comparing technologies with different production profiles,^8^ it is a relevant metric for comparing two technologies that would have similar generation output and value such as two peaker plants. Storage is increasingly planned and used as a resource for peak demand but evaluating its competitiveness is more challenging since it depends on the electricity price while charging, and as such we do not consider it in this section. For an anecdotal comparison, we have compared a levelized cost of storage, LCOS, to LCOE of gas plants in Figure S5. The LCOS, with variations for charging cost, is very similar to the Gas-CC/CT LCOEs indicating other operational factors could be defining in the selection between these technologies.

**Figure 1:**
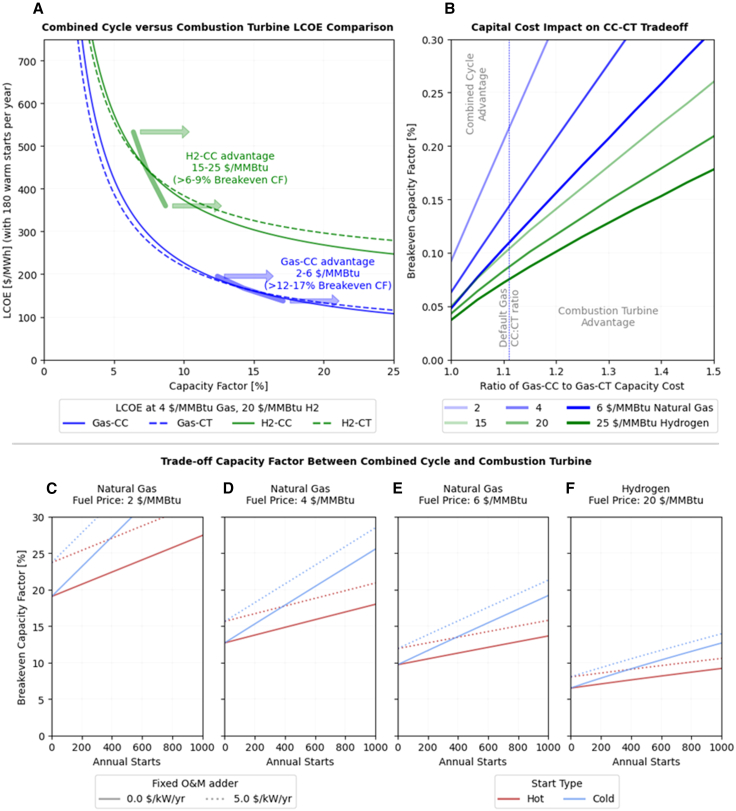
Analysis of economic trade-off point between CC and CT power plants based upon fuel price, capacity factor, and annual starts For a given fuel price and number of starts there is a capacity factor where CC and CT technologies will have the same levelized cost of energy, LCOE (A). The trade-off capacity factor depends upon the ratio of capital cost for the higher cost combined cycle to the combustion turbine, default is 1.1:1 (B) while all other cost components are directly from the ATB 2024.^7^ We extend our quantification of the trade-off point for a range of annual starts (1:1 CC to CT start), fixed O&M adders, and start type for 2, 4, and 6 $/MMBtu of natural gas (C–E) as well as 20 $/MMBtu ($2.25/kg) Hydrogen (F). These figures show the annual capacity factor at which the CT is more economically viable than the CC. Underlying costs are from ATB 2024^7^ for plants in 2035, reported in 2022$.

Figure 1 presents the LCOE estimates for CT and CCs at different annual capacity factors, as well as sensitivities to those values. In Figure 1A we compute LCOE curves for both plants using natural gas (assuming 4 $/MMBtu in 2022$) and for plants using an alternative fuel such as hydrogen (assuming 20 $/MMBtu, or about $2.25/kg hydrogen). As capacity factor decreases, the LCOE values increase nonlinearly, reflecting the amortization of the capital costs of the plant (which do not change based on usage) across fewer MWh of energy provided. Accordingly, CTs, (the dashed lines) which have lower capital costs, tend to have a lower LCOE and are more cost-effective than CCs (the solid lines) at very low capacity factors. For example, at 5% annual capacity factor, the LCOE for the natural gas CT (Gas-CT) is around 390 $/MWh, compared to nearly 425 $/MWh for a natural gas CC (Gas-CC).

At higher levels of utilization, fuel costs become more important, and the efficiency benefits of a CC begin to outweigh capital investment. The breakeven capacity factor at which point CCs are more economic than CTs occurs around 12%–17% for natural gas prices of 2$–6$/MMBtu, meaning that CTs tend to be more competitive as peakers for capacity factors below that threshold. With higher fuel prices, the breakeven capacity factor narrows and shifts to much lower values. Assuming 15$–25$/MMBtu for hydrogen fuel, the breakeven capacity factor below which CTs outcompete CCs falls to 6%–9%.

We explore the relative importance of capital cost and efficiency in determining peaker competitiveness in Figure 1B, which highlights the breakeven capacity factor as a function of the capital cost premium of a CC relative to a CT and the effective fuel cost faced by the plant. From our default natural gas assumptions (fuel costs: 4 $/MMBtu; capital costs: Gas-CC = 1.1× Gas-CT), an increase in Gas-CC capital cost of 10 percentage points to 1.2× the cost of a CT raises the lower bound breakeven capacity factor from 14% to 20%. In contrast, a similar breakeven increase occurs with a 50% increase in fuel costs to 6 $/MMBtu. For the higher fuel cost associated with hydrogen, there is a much larger area of capital cost and annual capacity factors at which CCs are the more economically competitive alternative. Reinforcing that with much higher fuel costs, efficiency plays a much stronger role relative to capital costs in determining if a peaker is economic.

Panels (a) and (b) of Figure 1 highlight the tradeoff between capital cost and efficiency. However, CCs that were designed as intermediate plants but take on more of peaking role might incur additional starts or cycling beyond their design, and therefore potentially face increased start or fixed operations and maintenance costs (for specific studies and the flexibility parameters used in these assumptions, see Table S3). To account for this, we explore how the breakeven capacity factor changes after incorporating CC start costs, using cost estimates for hot and cold starts, as well as higher fixed operations and maintenance costs (FOM). We do not assume an increase in Gas-CT FOM due to higher cycling as these plants are already high cycling and typically designed to be flexible. Modern CC plants can also be designed for more starts and cycling, which we do not explore here but discuss in the last section.

Intuitively, more frequent starts—particularly cold starts—as well as higher FOM costs erode the competitive advantages of CCs at low capacity factors, increasing the breakeven capacity factor between CTs and CCs (Figures 1C–1F). However, the relationship between start and FOM costs and the breakeven flattens as fuel costs increase. At 4 $/MMBtu, adding 1000 cold starts increases the annual capacity factor at which CCs are competitive from 13% to 26%; for hydrogen fuel at 20 $/MMBtu, those same starts only increase the breakeven from 7% to 13%. A $5/kW/yr FOM increase (on a $30.1/kW/yr base) would increase the breakeven by 2.5 percentage points at 6 $/MMBtu fuel costs and by 5 points at 2 $/MMBtu.

Future scenarios with substantially higher fuel costs—whether because of natural gas price increases from market or policy forces, or from switching to more expensive fuels—would shift the scale of impact further toward fuel cost than capital cost premium, favoring more efficient plants over less capital-intensive ones. Notably, there is a range at which the cost differences between the two are relatively small. For a capacity factor range of 11%–18% the LCOE values for Gas-CT and CC are within 2.5% of each other assuming 4 $/MMBtu gas (17%–27% for 2 $/MMBtu); for a hydrogen CT (H2-CT) and CC (H2-CC) the capacity factor range across which the LCOE difference is small is 5%–10%. Although these differences can be meaningful for utilities that need large amounts of peaking resources, they also suggest that in practice there may be overlap across different peaking options.

## What plants have historically served as peakers?

The peaking role is currently being filled predominantly by natural gas combustion turbines but a range of technologies, including some combined cycle, petroleum-, coal-fired plants have also historically operated with low utilization (<20% capacity factor) to serve that role.

At today’s natural gas prices, the above analysis suggests Gas-CTs are likely to be the most competitive option for peakers operating at less than 12%–17% annual capacity factor. In practice, however, system operators face a range of uncertain factors when making planning and operational decisions, which may result in a range of different plants operating in the peaking role.

**Figure 2:**
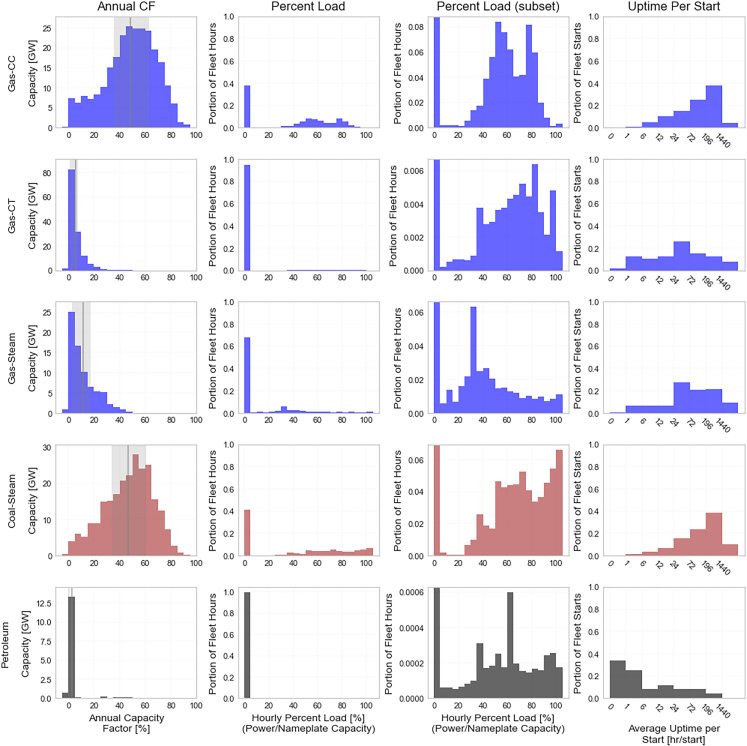
Distribution of thermal generation annual capacity factors (CF), percent load, and uptime per start Capacity factor data (left column) is calculated from EIA EIA 860^9^ and EIA 923^10^ for 2014–2023. The gray line indicates the median, while the shaded region indicates the interquartile range (25%–75%). Percent load (middle two columns) is the fraction of power output to the nameplate capacity; left-center shows full distribution, right-center is scaled to show the technology specific non-zero distribution. Uptime per start (right column), can characterize the commitment of the generator type (e.g., short peaks or seasonal). Percent load and uptime per start are calculated from EPA’s clean air markets program data (CAMPD) accessed through the public utility data liberation project (PUDL)^11^ for 2018–2022.

To understand what types of power plants have historically served as peaking plants, we use net generation and nameplate capacity data from the energy information administration to estimate annual capacity factor by generating unit for the last decade (2014–2023).^9^^,^^10^ This method allows for a heuristic categorization which is also available in the later long-term planning models (where hourly dispatches are not available) but does mean that a plant operating at high utilization for few hours may present the same as a plant operating at low utilization for long durations. The distributions of annual capacity factors for non-cogeneration fossil plants are summarized in the first column of Figure 2, with a wider range of thermal and storage capacity factors shown in Figure S1.

The capacity factor results in Figure 2 show that Gas-CTs tend to operate at low levels associated with peakers, with 96% of all Gas-CT operating below a 20% annual capacity factor. The distributions are similar for gas steam and petroleum plants (all mover types for petroleum combined, e.g., combustion turbine, internal combustion engine), with 79% and 95% of capacity operating below 20% annual capacity factor, respectively. In contrast, the distributions for Gas-CC and coal plants show much higher utilization, with medians between 45 and 50% capacity factor. Despite this, 11% of Gas-CC and 10% of coal capacity operate below 20% capacity factor, and there are instances of Gas-CT, gas steam, and petroleum plants operating as high as 40% capacity factor. Thus, although the economics of capital and fuel costs favor certain technologies as peakers, in practice there is overlap across resource types that play a peaking role. Plants have also exhibited trends over time; from 2014 to 2023, the percent of Gas-CC capacity operating as a peaker decreased from 17% to 6% while the median capacity factor increased from 42% to 56%. At the same time peaking coal declined from 39% to 18% of capacity but with declining median capacity factor, 59%–38% (Figure S3).

Though capacity factor provides a useful metric for identifying peaking resources, other operational characteristics can also shed light on how peakers are being used. To that end, we also analyze percent plant load—the percent of nameplate capacity the plant is dispatched when online—and uptime per start from 2018 to 2022 using the EPA’s clean air markets program data accessed through the public utility data liberation project.^11^ These data is reported at the hourly resolution and includes the operating time within each hour and load on the unit. To find the uptime per start, we sum the total operating hours for each unit and divide by the sum of hours in which operating time going from 0 to any non-zero value in a subsequent hour. At power plants with multiple units for the same generation process (i.e., a combined cycle power plant), the annual starts and uptimes were averaged across units.

When dispatched, gas-CC and coal plants tend to run at higher capacity factors and for longer continuous periods (Figure 2 columns 2–4). Plants operating at low percent load may also be reserving headroom to contribute operating reserves to this system, which we do not capture in these data. Although gas-CT are also dispatched at high-capacity levels, they tend to turn on and off more regularly, with most plants operating for less than 72 h at a time when on. In contrast, petroleum plants have very low annual capacity factors (3.1% average), tend to be dispatched at lower levels, and have shorter operating times when started (59% of starts have ≤6 h of uptime). This suggests a range of different peaking solutions, with gas-CTs and occasionally gas-CC or coal units filling in for seasonal, multi-day peaking requirements, and petroleum plants, which have the highest fuel and operating costs, being used only to meet the least frequent peaking events. Similar to the annual capacity results, the operational patterns of each plant type exhibit significant variability, indicating that a range of resource types can potentially serve peaking roles.

## What do models suggest about the future of peaking plants?

Future scenarios suggest peaking capacity might increase, in some cases doubling by 2050, but the generation resources providing this peaking capacity vary across scenarios. A diverse set of technologies capable of filling this role results in greater value for efficiency, yielding more gas-CC and battery storage. This is magnified in emission constrained scenarios which could swing toward combined cycle, hydrogen turbines, and battery storage to serve a critical peaker role in the future.

Increasing shares of wind and solar might increase the need for other resources to operate more like peakers to manage variability and meet peak load at time when those resources are not available. This intuitively results in three possible outcomes: an increase in the capacity of traditional peaking power plants (e.g., gas-CTs), a shift in operation of/investment in the current intermediate capacity (e.g., using existing gas-CC as a peaking resource rather than building new peakers), or a new technology becomes the peaking resource of choice in the future system (e.g., storage or hydrogen).

The decision of which path or combination thereof relies on many factors, such as fuel prices, technology costs, policies, availability of new technologies. It also relies on the ability of built resources to adapt to new operations, with this factor being one of the least captured. For example, systems seeking to reduce emissions may rely less on gas-CTs for peaking power due to their lower efficiency and higher per unit emissions, shifting other plant types into this role.

To provide one view on electricity system evolution, we draw on future buildouts published in the national renewable energy laboratory’s 2024 standard scenarios report.^12^ These scenarios are produced using the ReEDS capacity expansion model,^13^ which uses least cost linear optimization to determine the system buildout that satisfies key system constraints subject to input assumptions. The scenarios include a range of sensitivities, including technical, cost, and load growth assumptions as well as some “current policy” scenarios, some with existing regulations removed, and others meeting carbon emissions targets. In the emissions target scenarios, emitting sources can remain online but must be offset with negative emissions technologies, such as bioenergy-CCS (BECCS) or direct air capture (DAC) such that the system meets emission caps. Accordingly, these emissions reduction scenarios effectively increase the effective fuel cost for emitting sources, as the emissions cap forces the system to build and operate negative emissions to offset carbon emissions. These future-facing results do not consider some potential system charges such as aggressive demand-side changes, which might affect the deployment of peaking power plants.

**Figure 3:**
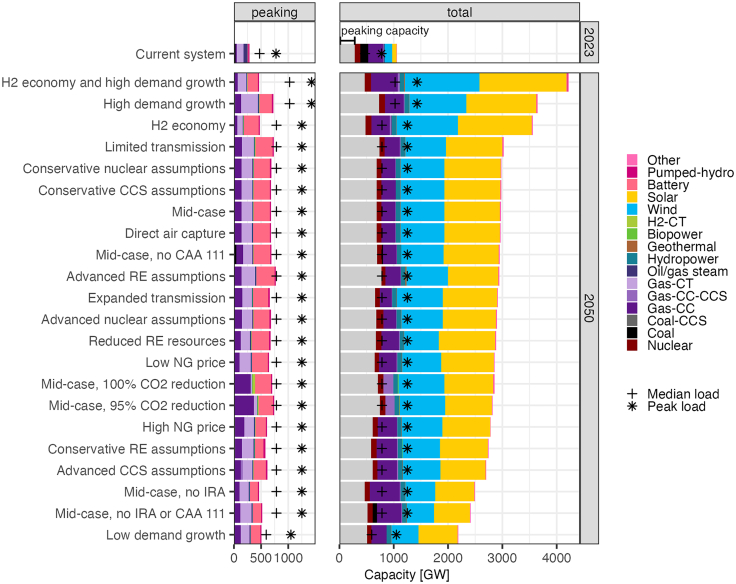
Installed peaking and non-peaking capacity in the 2023 system using 2023 EIA data^9^^,^^10^ and in future systems based on the 2024 standard scenarios^12^ Peaking capacity is defined as thermal or storage capacity below 20% capacity factor. In the total system column, the peaking capacity is aggregated into a light gray bar. Storage capacity factor calculated based on discharge. Median and peak load data are provided as a reference. “Other” includes Canadian imports and landfill gas plants.

For each scenario, we categorize the amount of peaking capacity, which we define as thermal or storage capacity operating at less than 20% annual capacity factor. This threshold value was selected as it captured at least 95% of traditional peaking resources such as gas-CTs and petroleum plants and only 10%–11% of intermediate plants such as gas-CC and coal. We acknowledge that this threshold is subjective, and arguments can be made for higher or lower bounds. This peaking definition is imperfect and, as outlined above, other factors such as cycling characteristics are important indicators of peaking as well. Embedding more of these dynamics is precluded by the available data generated using representative days, greater than hourly resolution, and linear dispatch values (rather than an integer commitment). Future work could use a unit commitment/production cost model on projections to build more robust characterization of the type of peaking behavior these plants display (e.g., seasonal peakers versus daily cycling peakers). Figure 3 shows the capacity fitting our peaking definition and overall system capacity in 2050 for 22 scenarios.

Across the scenarios, peaking capacity in 2050 (460–770 GW) is roughly double the approximately 280 GW of peaking capacity of today’s system. Although the total share of capacity used as peakers tends to fall with large deployments of wind and solar capacity, the share of non-VRE resources used as peakers increases. In today’s system, approximately one-third of thermal and storage capacity is used with less than 20% annual capacity factor, compared to a median value of 57% for the 2050 scenarios. The deployment of large amounts of low capacity factor VRE resources coupled with peaking resources seen here is consistent with other forward-looking studies; for example, in the Net Zero America study wind and solar provide 85%–90% of annual generation and are supported by 500–1000 GW of “firm” capacity across a range of scenarios.^14^

The increased deployment of peakers is driven by load growth coupled with the large projected increase in wind and solar, which provides the bulk of energy throughout the year in these scenarios but are unable to meet demand in every hour of the year. In nearly all cases, the quantity of thermal capacity stays similar to the current system or increases by 2050, but with less growth than the overall peaking capacity. Across all scenarios there is also a significant growth of battery storage, with 2050 deployments ranging from 140 to 340 GW relative about 13 GW in the 2023 system. Although renewable capacity varies widely across scenarios, the thermal peaking capacity is relatively consistent, suggesting a peaking saturation point.

In the current policy scenarios, most of the peaking capacity is provided by gas-CT units and storage, with minor contributions from gas-CC and coal. These coal plants are not cycled on and off like gas-CTs but run for several days at a time during high load periods, then are turned off for long periods until the next high-load event occurs. Overall, these patterns align with the historical data and the economic analysis of breakeven capacity factors from the previous sections.

For the 95% and 100% emissions reduction scenarios, most thermal peaking capacity is provided by natural gas. Due to the increased value of efficiency (driven by the emissions constraint), most of this capacity is gas-CC. In these scenarios, gas-CC is not used above 20% capacity factor unless coupled with CCS, highlighting the possibility of a significant operational shift in which gas-CC are used more extensively as peakers. In these scenarios, the amount of gas-CC capacity used as peaking capacity exceeds the total gas-CC on the current system. The 100% emissions reduction scenario also deploys H2-CTs as a peakers, using both new builds and retrofits from existing gas-CT plants (H2-CCs are not available in this model). Both investments reflect the increased implicit cost on the combustion of natural gas, which would need to be offset with relatively expensive negative emissions. Although emission constrained scenarios also deploy gas-CC with CCS, the high capital cost for these plants means they are not utilized as peaking resources. The mix of storage, gas-CC, gas-CT, and H2-CT deployed reflects the competition across different capital and fuel costs, with factors, such as VRE availability, transmission, availability of biomass for BECCS, and the cost of DAC affecting how these resources interact to providing peaking in the future.

Presumably, some of the peaking power needed in the emissions constrained scenarios could come from existing plants. For example, the current grid has approximately 284 GW of gas-CC capacity, compared to an average of 300–650 GW shown in 2050 in these future scenarios, with 50–370 GW of that capacity operating as a peaker. Retrofitting existing gas-CC or changing their operational patterns from intermediate, load following plants to peakers could potentially help meet future peaking requirements at reduced cost. Although the historical analysis suggests that there is a meaningful amount of gas-CC capacity already operating at low capacity factor, there is uncertainty as to whether today’s fleet can provide the peaking services required for the future system.

## Conclusions and research needs for planning

More research is needed to understand how the peaking capacity role might be filled by new resources, such as combined cycle plants using natural gas or alternative fuels such as hydrogen. And further work is needed to understand if these resources are adequately represented in these long term planning models.

Peakers have always been an important component of reliable and cost-effective power systems, and the analysis in the previous section suggests this is likely to be the case under a wide range of future conditions. Across the future scenarios studied, there is a need for the traditional peaker plant, though the fuel source and unit type for that peaker might evolve. Both the current economic analysis and the future scenarios suggest that there are conditions under which gas-CC might become more competitive as a flexible peaking resource. Most influential in this shift toward increasing portions of gas-CC operating at low capacity factors are high gas prices and emission constraints, but in all scenarios, short of those with hydrogen economies, more gas-CC operates lower than 20% both absolutely and in proportion to total gas-CC capacity. Especially in emission constrained power systems, the modeling shows that gas-CC may become primarily a peaking resource. One reason for this shift is the increase in zero-marginal-cost generators which can reduce the number of hours where an intermediate fuel-based generator is needed. These factors are well represented in current models, but the plant-level and long-term planning impacts of this operational change on the gas-CC plants and other intermediate resources are not.

When comparing historical to modeled results, there are many uncertainties which come into play such as the ability of technologies to change parameters as they develop (e.g., lower learned costs for storage, increased flexibility of new thermal power plants, challenges of power plant siting). There is a potential for storage to displace the niche for thermal peaking power plants and the model results considered in this study do highlight the growth of storage as a peaking resource as we define it. Even in this context, cases with advanced renewable and storage costs show an increase in thermal peaking capacity (labeled *Advanced RE Assumptions* in Figure 3). With this growth of peaking gas-CC as a portion of total gas-CC capacity across nearly all studied scenarios, it is necessary to highlight the divergence from plant precedent in the U.S. power system. It should be considered how much this discrepancy is due to technology change, operational change, or just an area where planning models could improve.

There is a trend toward deployment of storage^15^ and increased discussion of demand side resources to serve changing peak trends.^16^ Keskar et al.^16^ highlights how potential winter peak growth could be best served by non-generation methods, in one case highlighting the tensions around Duke Energy Carolinas IRP gas investment plan. Even with the pressure noted in this article and potentially less expensive options, in later IRPs Duke Energy Carolinas planned for even more gas-CTs while also expecting significant storage growth.^17^

In carbon emission-constrained scenarios, we found gas-CC capacity in 2050 exceeded the total gas-CC installed today, suggesting not only the need for new gas-CC plants, but also the importance of existing gas-CC transitioning to a peaking role. Although the historical analysis suggests a sizable portion of gas-CCs operate with low annual capacity factors, these plants typically exhibit a lower efficiency (Figure S4). It is unclear if lower efficiency causes the lower utilization or vice versa, thus making it unclear whether most existing gas-CC plants can achieve this flexibility without significant operational changes, efficiency penalties, or maintenance increases (for specific referenced impacts, see Table S3). While a gas-CC can operate its gas turbine alone (thus behaving as a gas-CT), this capability is not represented in our analysis and could be an important aspect for future research and for planning models to consider.

In the US power system, gas-CT can be seen as an archetypal peaking plant and gas-CC as an intermediate generator. Due to this prevalence the empirical and accessible data on high cycling domestic gas-CCs is limited. In an analysis performed for the Irish power system, where the islanded system and increased VRE has induced high cycling to thermal generators, O&M costs were seen to correlate with increases in cycling.^18^ These costs were highest for older conventional steam power plants, but it is suggested that gas-CC plants could be exhibiting this general trend just on newer plants. This aligns with potential impacts to nearly all components of the combined cycle plant by Black and Veatch^19^ (e.g., reduced efficiency, increased outages, increased O&M costs).

Given these considerations, treating intermediate resources in a more nuanced manner in planning models would better reflect how their operational changes are likely to impact their performance. One option would be reparameterization including variable outage rates, O&M costs, and heat rate adjustments given the cycling or capacity factor of modeled technologies. Planners might also consider uncertainty in peaking requirements across a range of future scenarios to assess their own needs with regard to the intermediate-to-peaking resource transition. Both methods could be computationally challenging and would require more detailed data of these power plants.

As has been shown, planning models indicate that peaking will continue to be a critical element of the future power system, with the potential for a wider mix of resources serving in the peaking capacity role. Although in the U.S. Gas-CTs have long fulfilled this role because of their relatively low capital costs, some factors may result in gas-CCs and other resources becoming increasingly operated as peakers. There is a wide range of uncertainty in both the technical and economic viability of a wide range of non-CT peaking options, including storage, non-variable renewables such as geothermal or biomass, hydrogen fuel cells, advanced nuclear, and plants with carbon capture and sequestration. While this perspective highlights that there will likely always be a role for low-capital cost, high marginal cost peakers, planners will need to evaluate changes to their systems and to peaking operations more generally when weighing the mix of technologies to deploy to meet their peaking needs.

## Data and code availability

All figure producing code and some processed data (if not from direct sources) is available at https://doi.org/10.5281/zenodo.18174127. No new data were produced in the production of this perspective.

## Acknowledgments

This work was authored by the National Laboratory of the Rockies for the U.S. Department of Energy (DOE) under contract no. DE-AC36-08GO28308. Funding provided by the Strategic Analysis Team. The views expressed in the article do not necessarily represent the views of the DOE or the U.S. Government. The U.S. Government retains and the publisher, by accepting the article for publication, acknowledges that the U.S. Government retains a nonexclusive, paid-up, irrevocable, worldwide license to publish or reproduce the published form of this work, or allow others to do so, for U.S. Government purposes. We also thank Ashna Aggarwal, Kate Anderson, Sam Baldwin, Gregory Brinkman, Patrick Bryant, Jaquelin Cochran, Stuart Cohen, Victor Duraes de Faria, Gian Porro, and Mark Ruth for their feedback and reviews.

## Author contributions

Methodology, formal analysis, writing - original draft, writing - review & editing, visualization, M.V. conceptualization, methodology, formal analysis, writing - original draft, writing - review & editing, visualization, project administration, B.S. conceptualization, methodology, writing - original draft, writing - review & editing, supervision, project administration, funding acquisition, W.C. conceptualization, supervision, P.D. conceptualization, writing - review & editing, supervision, T.M.

## Declaration of interests

The authors declare no competing interests.
