# Supplementary material for: The past, present, and future of peaking thermal power plants in the United States
Source: iScience. 2026 Feb 2;29(3):114886. doi: 10.1016/j.isci.2026.114886 (PMC12925234; doi:10.1016/j.isci.2026.114886)
Supplement: Document S1. Figures S1–S5 and Tables S1–S3 [file mmc1.pdf]

iScience, Volume *29*

Supplemental information

The past, present, and future of peaking thermal power plants in the United States

Max Vanatta, Brian Sergi, Wesley Cole, Paul Denholm, and Trieu Mai

Supplemental Information

Supplemental methods

Levelized cost of energy (LCOE) calculations

For this work, we calculate a simple LCOE using the following equation:

$$LCOE = \frac{(CC \times CRF) + FOM + (Starts \times SC)}{CF \times 8760} + VOM + (HR \times FP)$$

Where *LCOE* = levelized cost of energy [$/MWh], *CC* = capital cost [$/MW], *CRF* = capital recovery factor [dimensionless], *FOM* = fixed operations and maintenance [$/MW], *Starts* = number of annual starts [dimensionless], *SC* = start cost [$/MW/start], *CF* = annual capacity factor [%], *VOM* = variable operations and maintenance [$/MWh], *HR* = heat rate [MMBTu/MWh], *FP* = fuel price [$/MMBTu]. 8760 represents the number of hours in a non-leap year for an annual LCOE (to align with the annualization of the CRF). Parameters used for this calculation are in *SI table 1*.

The CRF for this work is calculated assuming a book lifetime (*lt*) of 20 years and a discount rate (*DR*) of 8% using the following equation:

$$CRF = \frac{DR}{1 - \left(\frac{1}{(1 + DR)^{lt}}\right)}$$

SI Table 1: Parameters used for levelized cost of energy, LCOE, calculations.

	**Gas-CC**	**Gas-CT**	**H_2-CC**	**H_2-CT**	**Li-Ion Battery**	**Source**
Capital Cost [$/kW]	1,312	1,182	1,307	1,218	306 (power)<br>223 (energy)	2024 NREL ATB[3]
Fixed Operations and Maintenance [$/kW/yr]	30.1	24.2	29.0	24.2	7.66 (power)<br>5.58 (energy)	2024 NREL ATB[3]
Variable Operations and Maintenance [$/MWh]	1.96	6.95	3.73	6.95	--	2024 NREL ATB[3]
Start Cost [$/MW/start] (Hot, Warm, Cold)	31, 44, 60	22, 28, 38	31, 44, 60	22, 28, 38	--	Kumar et al.[4]
Heat Rate [MMBtu/MWh]	6.159	9.717	7.565	9.717	0.85 (rte)	2024 NREL ATB[3]

Levelized cost of storage (LCOS) calculations

For a comparison of storage to gas power plants, we perform a quick comparison using a rough LCOS of lithium-ion battery storage. We calculate the LCOS with the following equation:

$$LCOS = \frac{(CC_{power} + CC_{energy} \times EP) \times CRF + (FOM_{power} + FOM_{energy} \times EP)}{8760 * CF} + \frac{CP}{rte}$$

Where capital costs, *CC*, and fixed O&M, *FOM*, are allocated to energy and power components, *EP* = energy to power ratio assumed to be 4, *CP* = charging price [$/MWh], and *rte* = round trip efficiency [%]. For the input values we use NREL ATB values (above) and a lifetime of 15 years.

Supplemental figures and tables

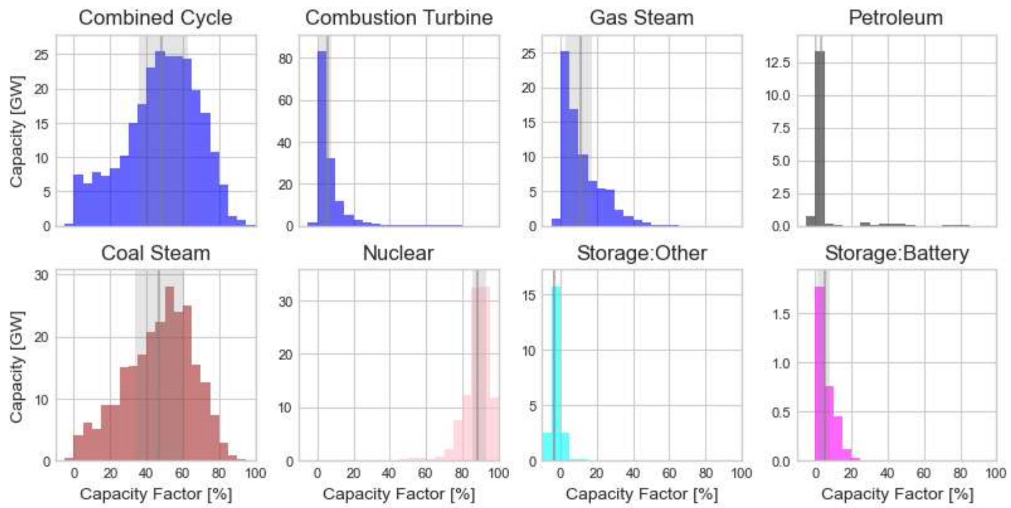

SI Figure 1: Annual capacity factor distribution for thermal+ storage plant types. Each distribution has a solid line for the median value of the set and a shaded region for the interquartile range. Capacity factor data is calculated from EIA 860[1] and EIA 923[2] for 2014-2023.

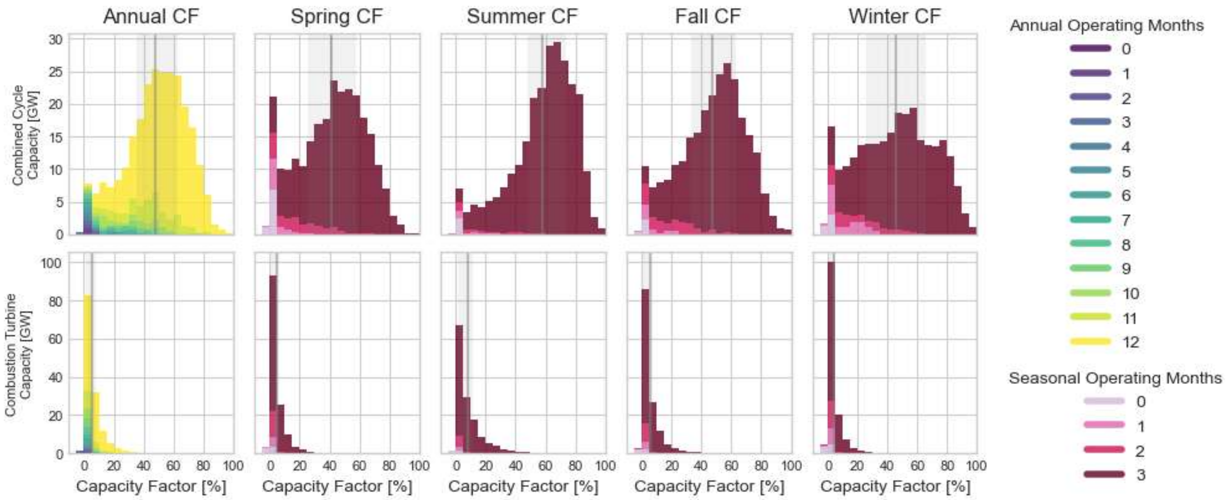

SI Figure 2: Annual and seasonal distribution of capacity factors for Gas-CC and Gas-CT. The distribution color represents the number of months during the labeled time period when the plant generates power. Each distribution has a solid line for the median value of the set and a shaded region for the interquartile range. Capacity factor data is calculated from EIA 860[1] and EIA 923[2] for 2014-2023.

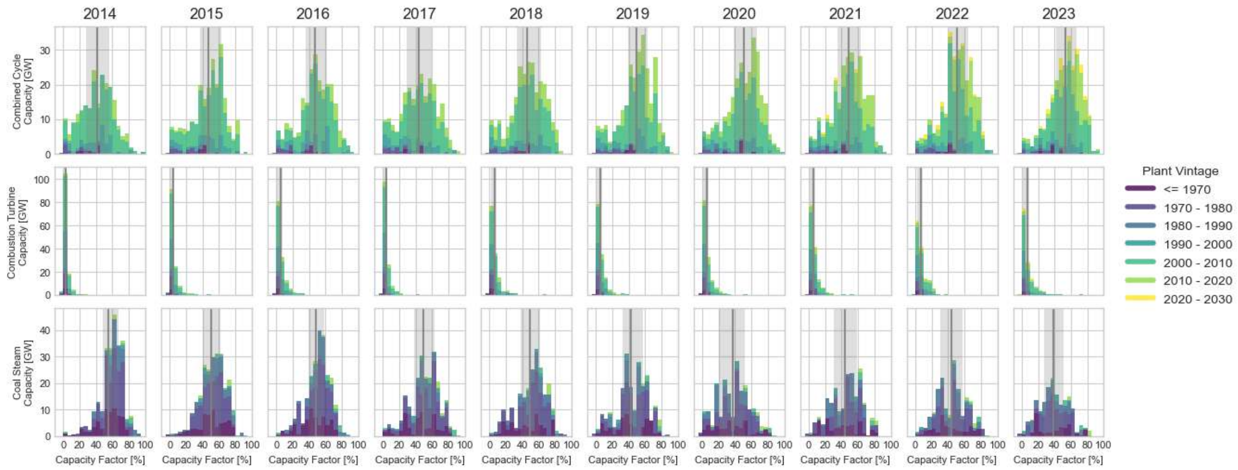

SI Figure 3: Annual capacity factor distribution across years and by plant vintage. Each distirbution has a solid line for the median value of the set and a shaded region for the interquartile range. Capacity factor data is calculated from EIA 860[1] and EIA 923[2] for 2014-2023.

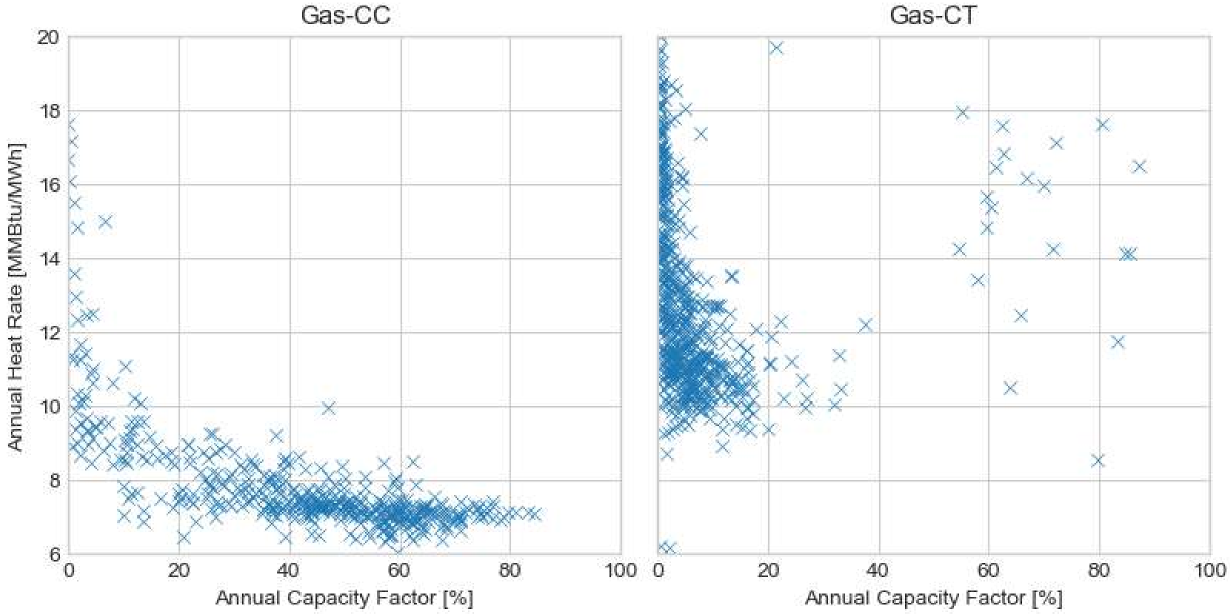

SI Figure 4: Relationship between the mean annual capacity factor and mean annual heat rate for Gas-CC and CT plants. Capacity factor and heat rate data are calculated from EIA 860[1] and EIA 923[2] for 2014-2023. Heat rate is calculated as the reported fuel used for electricity divided by the annual net generation as reported to the EIA.

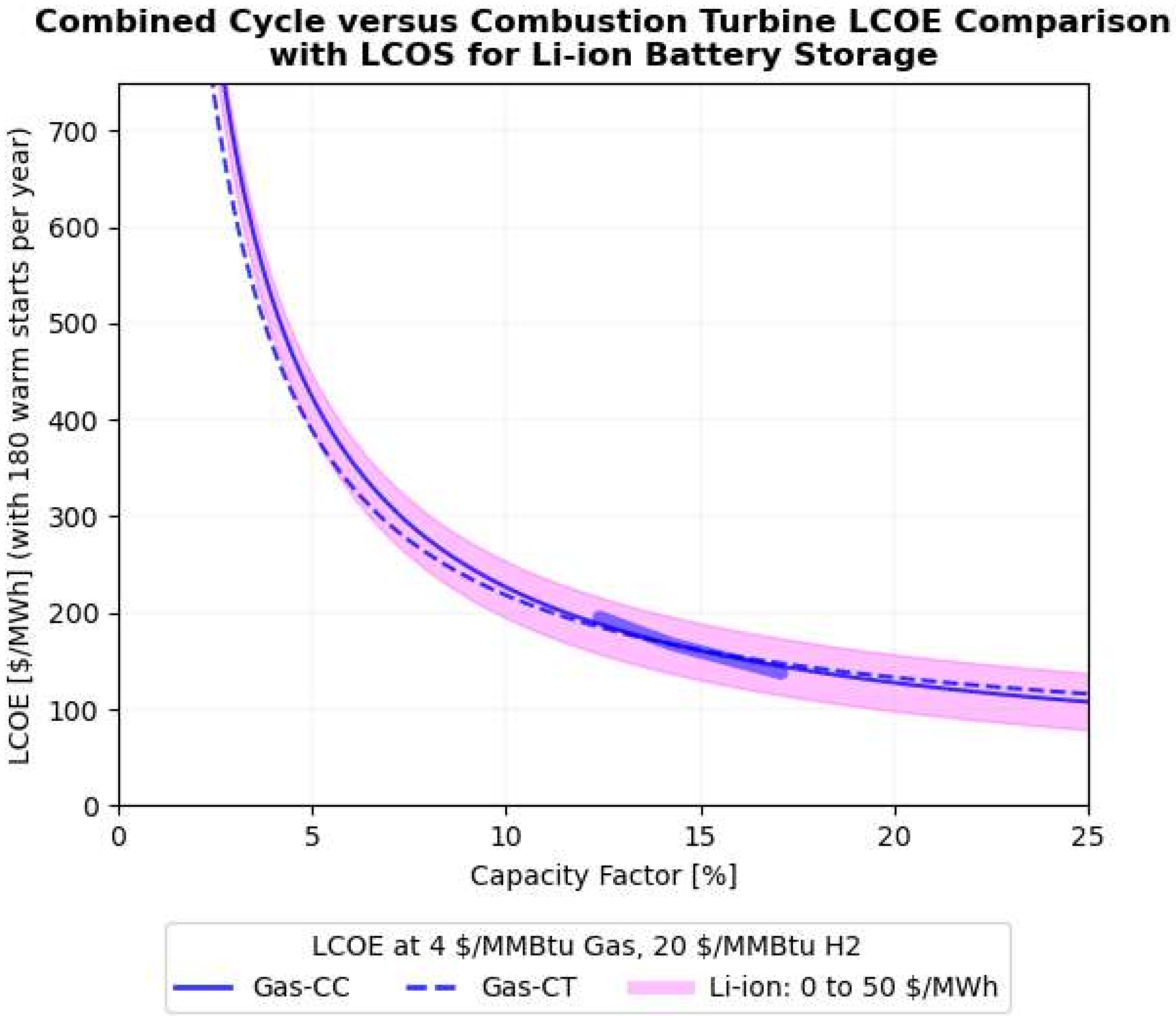

SI Figure 5: Comparison of LCOE and LCOS for gas and li-ion battery storage across capacity factors. Range of values for battery storage is the range for charging costs from 0 to 50 $/MWh across the year.

SI Table 2: Standard Scenario Descriptions.

Name	Description
Mid-case, current policies	A business-as-usual scenario. Represents current policies.
Mid-case, 100% by 2035 decarb	A scenario with the same assumptions as the Mid-case, except for an assumed policy that enforces 100% electric-sector decarbonization by 2035.
Mid-case, 95% by 2050 decarb	A scenario with the same assumptions as the Mid-case, except for an assumed policy that enforces 95% electric-sector decarbonization by 2050.
Direct air capture, current policies	A scenario with the same assumptions as the Mid-case, except for including electricity-powered direct air capture. Represents current policies.
Conservative CCS assumptions, current policies	A scenario with the same assumptions as the Mid-case, except for conservative assumptions of CCS cost and performance. Represents current policies.

High demand growth, current policies	A scenario with the same assumptions as the Mid-case, but with higher demand growth representing significant electrification. Represents current policies.
Hydrogen economy, current policies	A scenario with the same assumptions as the Mid-case, but with exogenous hydrogen demand. Represents current policies.
Hydrogen economy and high demand growth, current policies	A scenario with the same assumptions as the Mid-case, but with exogenous hydrogen demand and higher demand growth representing significant electrification. Represents current policies.
High NG price, current policies	A scenario with the same assumptions as the Mid-case, but with higher natural gas prices. Represents current policies.
Conservative RE assumptions, current policies	A scenario with the same assumptions as the Mid-case, except for conservative assumptions of renewable energy cost and performance. Represents current policies.
Limited transmission, current policies	A scenario with the same assumptions as the Mid-case, except for limitations on transmission expansion. Represents current policies.
Advanced CCS assumptions, current policies	A scenario with the same assumptions as the Mid-case, but with advanced assumptions for CCS cost and performance. Represents current policies.
Low demand growth, current policies	A scenario with the same assumptions as the Mid-case, but with lower demand growth. Represents current policies.
Low NG price, current policies	A scenario with the same assumptions as the Mid-case, but with lower natural gas prices. Represents current policies.
Advanced nuclear assumptions, current policies	A scenario with the same assumptions as the Mid-case, but with advanced assumptions for nuclear cost and performance. Represents current policies.
Advanced RE assumptions, current policies	A scenario with the same assumptions as the Mid-case, except for advanced assumptions of RE cost and performance. Represents current policies.
Reduced renewable energy resources, current policies	A scenario with the same assumptions as the Mid-case, except for an assumption of less area available for renewable energy development. Represents current policies.
Conservative nuclear assumptions, current policies	A scenario with the same assumptions as the Mid-case, but with conservative assumptions of nuclear cost and performance. Represents current policies.
Mid-case, No Clean Air Act 111	A scenario with the same assumptions as the Mid-case, except that there is no representation of the updated Clean Air Act 111 rules.
Mid-case, No Inflation Reduction Act or Clean Air Act 111	A scenario with the same assumptions as the Mid-case, except that there is no representation of the updated Clean Air Act 111 rules or the electric-sector IRA tax credits.
Mid-case, No Inflation Reduction Act	A scenario with the same assumptions as the Mid-case, except that the electric-sector IRA tax credits are not represented.
Expanded transmission, current policies	A scenario with the same assumptions as the Mid-case, but with VSC HVDC transmission lines enabled as investment option.

SI Table 3: Additional resources and descriptions of the factors underlying the main text analysis.

Parameter, assumption, or dataset	Description	Source
Start & cycling costs	Cost per start (across cold, warm and hot) for the technologies included.	Kumar et al.[4]
Impacts of increased cycling	Descriptions and quantifications of higher cycling impacts using the Irish power system. Cycling increases the O&M costs incurred by Gas-CCs.	Keatley et al. [5]
	Heat rates, O&M costs, and outages are likely to increase with increased cycling without deliberate effort.	Muggli and Knowling [6]
	Values for the increased costs and sources of cycling impacts on Gas-CC plants.	EPRI [7]
	Industry magazine description of the current challenges and options for how to mitigate the Gas-CC cycling concerns.	Espinoza et al. [8]
Natural gas projected costs	The range of natural gas prices explored in the main text is roughly consistent with EIA's 2023 AEO reference projection of natural gas price, which falls between 3-4 $/MMBtu through 2050.	EIA's 2023 AEO [9]
EIA-EPA crosswalk	The EIA and EPA datasets do not inherently have the same unit definition and therefore we connect these using the EPA's crosswalk.	EPA's crosswalk[10]
EPA data retrieved from PUDL	Plant load and uptime per start from the EPA's Clean Air Markets Program Data accessed through the Public Utility Data Liberation Project[11].	EPA's Clean Air Markets Program Data[12]

SI references

1. U.S. Energy Information Administration (EIA) (2024). Annual Electric Power Industry Report, Form EIA-860 detailed data with previous form data (EIA-860A/860B). https://www.eia.gov/electricity/data/eia860/index.php.

2. U.S. Energy Information Administration (EIA) (2024). Form EIA-923 detailed data with previous form data (EIA-906/920). https://www.eia.gov/electricity/data/eia923/index.php.

3. NREL (2024). 2024 Annual Technology Baseline (National Renewable Energy Laboratory).

4. Kumar, N., Besuner, P., Lefton, S., Agan, D., and Hilleman, D. (2012). Power Plant Cycling Costs https://doi.org/10.2172/1046269.

5. Keatley, P., Shibli, A., and Hewitt, N.J. (2013). Estimating power plant start costs in cyclic operation. Applied Energy *111*, 550–557. https://doi.org/10.1016/j.apenergy.2013.05.033.

6. Muggli, A., and Nowling, U. Flexible Operation of Combined Cycle Power Plants.

7. EPRI (2013). Impact of Cycling on the Operation and Maintenance Cost of Conventional and Combined-Cycle Power Plants.

8. Espinoza, N., Carson, B., and Roberts, R. (2014). Managing the Changing Profile of a Combined Cycle Plant. Power Magazine.

9. EIA (2023). Annual Energy Outlook 2023 (U.S. Energy Information Administration).

10. Tafoya, J., Ibarra, M., Schreifels, J., and Huetteman, J. (2022). EPA—EIA Power Sector Data Crosswalk. https://github.com/USEPA/camd-eia-crosswalk/blob/master/epa_eia_crosswalk.csv.

11. Catalyst Cooperative (2024). Public Utility Data Liberation Project.

12. United States Environmental Protection Agency (EPA) (2024). Clean Air Markets Program Data. (Office of Atmospheric Protection, Clean Air and Power Division).
